# Supplementary material for: Causal Relationship between Aging and Anorexia Nervosa: A White-Matter-Microstructure-Mediated Mendelian Randomization Analysis
Source: Biomedicines. 2024 Aug 16;12(8):1874. doi: 10.3390/biomedicines12081874 (PMC11351342; doi:10.3390/biomedicines12081874)
Supplement: Supplementary file 1 [file biomedicines-12-01874-s001.zip › Supplementary Figure/Figure S8/Leaveoneout plot of MR analysis for TL to ubm-b-2120.pdf]

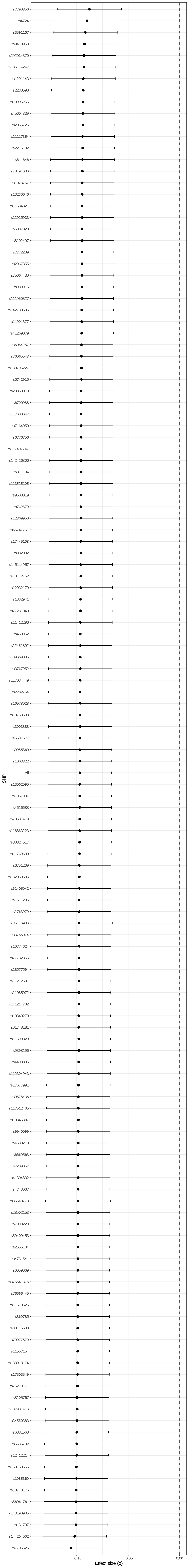

rs7790856
rs4724
rs3891167
rs9419958
rs202034370
rs185174247
rs1291143
rs2230590
rs10905255
rs45604339
rs2056726
rs11117354
rs2276182
rs611646
rs78491606
rs1023767
rs13230646
rs11584821
rs12925933
rs6007020
rs8102497
rs7772289
rs2967355
rs75664430
rs939916
rs111950327
rs142730696
rs11991877
rs41269079
rs6054257
rs76065543
rs139795227
rs5742915
rs28363070
rs6790988
rs117630647
rs7164950
rs6776756
rs117407747
rs142426306
rs871134
rs113525195
rs9600019
rs762679
rs12369950
rs55747751
rs17445108
rs932002
rs145114957
rs10112752
rs12932179
rs1332941
rs77231040
rs11412296
rs450962
rs12451892
rs139669835
rs3767952
rs117034449
rs2282764
rs16978028
rs10768683
rs3093888
rs6587577
rs9955360
rs1003322
All
rs13062095
rs1957937
rs4616688
rs73581419
rs116863223
rs80324517
rs11769630
rs6751209
rs182059586
rs61405042
rs1611236
rs2763979
rs35446936
rs3785074
rs10774624
rs77732866
rs28577594
rs11212631
rs11085072
rs141214782
rs10840270
rs61748181
rs11699829
rs9398196
rs4498805
rs112394943
rs17677991
rs9878436
rs117512405
rs10845387
rs9940099
rs4530278
rs6669563
rs7209057
rs41304832
rs4743037
rs35640778
rs28502153
rs7099229
rs59409453
rs2555104
rs4731541
rs6659669
rs376641875
rs76666449
rs11579626
rs869785
rs80116508
rs79977579
rs11557154
rs188918174
rs17803849
rs76219171
rs8105767
rs137901416
rs34550383
rs6881568
rs6536702
rs12412214
rs150150565
rs1985369
rs10773176
rs56061761
rs143190905
rs131797
rs144204502
rs7705526
SNP
−0.10
−0.05
0.00
Effect size (b)
